# Impact of the time interval between primary or interval surgery and adjuvant chemotherapy in ovarian cancer patients

**DOI:** 10.3389/fonc.2023.1221096

**Published:** 2023-08-16

**Authors:** Alberto Farolfi, Elisabetta Petracci, Giorgia Gurioli, Gianluca Tedaldi, Claudia Casanova, Valentina Arcangeli, Andrea Amadori, Marta Rosati, Marco Stefanetti, Salvatore Luca Burgio, Maria Concetta Cursano, Cristian Lolli, Valentina Zampiga, Ilaria Cangini, Giuseppe Schepisi, Ugo De Giorgi

**Affiliations:** ^1^ Department of Medical Oncology, IRCCS Istituto Romagnolo per lo Studio dei Tumori (IRST) “Dino Amadori”, Meldola, Italy; ^2^ Biostatistics and Clinical Trials Unit, IRCCS Istituto Romagnolo per lo Studio dei Tumori (IRST) “Dino Amadori”, Meldola, Italy; ^3^ Biosciences Laboratory, IRCCS Istituto Romagnolo per lo Studio dei Tumori (IRST) “Dino Amadori”, Meldola, Italy; ^4^ Oncology Department, Santa Maria delle Croci Hospital, Ravenna, Italy; ^5^ Romagna Cancer Registry, IRCCS Istituto Romagnolo per lo Studio dei Tumori (IRST) “Dino Amadori”, Meldola, Italy; ^6^ Department of Gynaecology and Obstetrics, Morgagni-Pierantoni Hospital, Forlì, Italy; ^7^ Department of Medical Oncology, Infermi Hospital, Rimini, Italy; ^8^ Department of Gynaecology and Obstetrics, Infermi Hospital, Rimini, Italy

**Keywords:** BRCA1/2 mutation, interval debulking surgery, ovarian cancer prognosis, primary debulking surgery, residual disease, time to initiation of chemotherapy

## Abstract

**Introduction:**

Primary debulking surgery (PDS), interval debulking surgery (IDS), and platinum-based chemotherapy are the current standard treatments for advanced ovarian cancer (OC). The time to initiation of adjuvant chemotherapy (TTC) could influence patient outcomes.

**Methods:**

We conducted a multicenter retrospective cohort study of advanced (International Federation of Gynecology and Obstetrics (FIGO) stage III or IV) OC treated between 2014 and 2018 to assess progression-free survival (PFS) and overall survival (OS) in relation to TTC. All patients underwent a germline multigene panel for BRCA1/2 evaluation.

**Results:**

Among the 83 patients who underwent PDS, a TTC ≥ 60 days was associated with a shorter PFS (hazard ratio (HR) 2.02, 95% confidence interval (CI) 1.04–3.93, *p* = 0.038), although this association lost statistical significance when adjusting for residual disease (HR 1.52, 95% CI 0.75–3.06, *p* = 0.244, for TTC and HR 2.73, 95% CI 1.50–4.96, *p* = 0.001, for residual disease). Among 52 IDS patients, we found no evidence of an association between TTC and clinical outcomes. Ascites, type of chemotherapy, or germline BRCA1/2 mutational status did not influence TTC and were not associated with clinical outcomes in PDS or IDS patients.

**Discussion:**

In conclusion, longer TTC seems to negatively affect prognosis in patients undergoing PDS, especially those with residual disease.

## 1 Introduction

The diagnosis of ovarian cancer is often at advanced stages due to the lack of an effective screening method and unspecific symptoms (1). The current standard of treatment consists of primary debulking surgery (PDS) aimed at complete macroscopic removal of the disease, followed by platinum-based consolidation chemotherapy (2). However, complete gross tumor resection may be difficult to achieve or to accept by the patients due to the consequence of surgery. For this reason, surgery may be postponed after having achieved a tumor response. Indeed, patients treated with neoadjuvant chemotherapy and interval debulking surgery (IDS) followed by consolidation chemotherapy have superimposable survival outcomes when compared to patients treated with PDS followed by consolidation chemotherapy in advanced epithelial ovarian with high tumor load (3).

Although the optimal time interval between surgery and initiation of chemotherapy in ovarian cancer remains unclear (4), it is highly probable that as a significant delay in initiating adjuvant therapy increases, so does the risk of a reduced benefit in clinical outcomes. So far, residual-tumor after surgery, International Federation of Gynecology and Obstetrics (FIGO) stage, type of surgery (PDS or IDS), tumor grade, and histological subtype are well-established prognostic factors (5). Age, performance status, presence of ascites, and genetic tumor characteristics were suggested as other possible predictors of clinical outcomes (5, 6). In this context, from a biological point of view, a longer time to initiation of chemotherapy (TTC) would seem detrimental, even if this issue has not been subjected to randomized controlled clinical trials. The results of retrospective studies on the relationship between TTC and survival outcomes have been controversial, particularly for ovarian cancer patients with residual disease after surgery (7–9).

Most clinicians, however, assume that adjuvant chemotherapy should commence as soon as possible after both PDS and IDS. This hypothesis is supported by analogies from other neoplasms (i.e., breast and colon cancer) where increased mortality is demonstrated among patients with longer TTC (10, 11).

However, ovarian cancer, more than malignancies originating from other organs, often requires advanced surgical procedures such as bowel resection, stripping, and diaphragm or liver resections in order to achieve complete gross tumor resection. However, multivisceral resections and increasing surgical complexity are associated with postoperative complications, which may prolong recovery and delay the initiation of consolidation chemotherapy (12), potentially influencing clinical outcomes. Thus, the aim of the present study was to investigate the effect of TTC in patients with advanced ovarian cancer after PDS or IDS on survival endpoints.

## 2 Materials and methods

Patients with a diagnosis of ovarian cancer referring to the IRST Genetic Counseling service or the Oncology units of the Area Vasta Romagna (AVR) catchment area in the years 2014–2018 were included in this analysis, as specified in our previous study (13). The study was performed in accordance with the Good Clinical Practice and the Declaration of Helsinki and approved by the AVR Ethics Committee (protocol 6326/2020). All the patients enrolled in the study have signed informed consent for the genetic analyses and the use of the results for research purposes.

**Figure 1:**
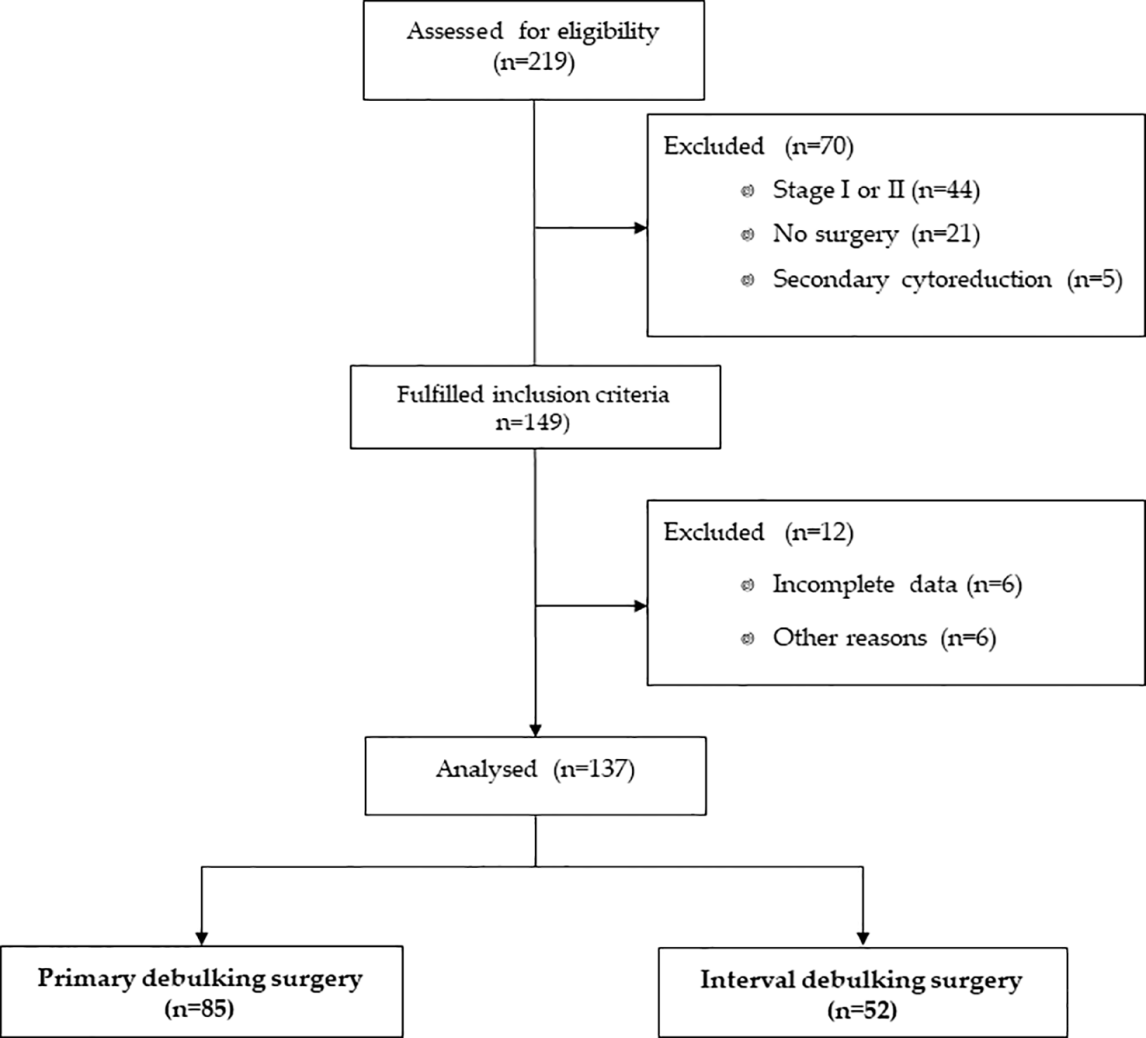
Flow diagram of patients included in the analysis.

For this analysis, we excluded patients who did not undergo surgery or underwent secondary cytoreductions. Patients with a FIGO stage I or stage II disease were also excluded. **Figure 1** represents the flow diagram of ovarian cancer patients included in this analysis.

Patient characteristics were summarized by means of mean ± standard deviation (SD), minimum and maximum values or median, and first and third quartile for continuous variables and by natural frequencies and percentages for categorical ones.

All the patients included in our analyses underwent a germline multigene panel to evaluate BRCA status. Mutational status was defined as specified in our previous work (13). In summary, patients were sequenced using a panel that analyzed 94 genes involved in hereditary cancer including BRCA1 and BRCA2 genes. Genetic variants were classified according to International Agency for Research on Cancer (IARC) recommendations (14) for BRCA1/2 variants and according to ClinVar (15) and dbSNP (16) for the other genes. Thus, patients were classified into three categories: germline BRCA1/2 mutant patients (BRCAmut), patients with pathogenic germline mutations in other genes (other mut), or germline wild-type (WT) patients.

TTC was calculated as the time, in days, from the definitive surgery to the initiation of adjuvant chemotherapy. Definitive surgery was considered the last surgical treatment with curative intent for ovarian cancer. For survival analyses, cases were divided at the 75th percentile of the interval from surgery to the start of chemotherapy into two groups (defined as early versus late start of chemotherapy), rounded at 60 days. In addition to the 75th percentile, TTC was also analyzed as a continuous variable.

The association between categorical variables and the type of surgery was tested by Pearson’s χ^2^ test or Fisher’s exact test, when appropriate, whereas those with a continuous variable were tested by means of Student’s t-test or analogous non-parametric Wilcoxon–Mann– Whitney test, when appropriate.

Progression-free survival (PFS) was defined as the time, in months, from the start of chemotherapy to disease progression or death from any cause, whichever occurred first. Overall survival (OS) was defined as the time, in months, from the start of adjuvant chemotherapy to death from any cause. Patients not experiencing the events of interest were censored at the most recent contact.

PFS and OS functions were estimated using the Kaplan–Meier method, and the log-rank test was used to assess differences between groups. Median PFS and OS were reported as point estimates and 95% confidence intervals (CIs). The Cox proportional hazards regression model was used to quantify the association between specific covariates and the time-to-event endpoints. Results are reported as hazard ratio (HR) and 95% CIs. All statistical analyses were performed using STATA 15.0 software (College Station, TX, USA).

## 3 Results

### 3.1 Clinico-pathological features

TTC information was available in 137 of the 219 patients enrolled in the previous study (**Figure 1**). Approximately two-thirds (n = 85, 62.0%) of patients underwent PDS, while 52 patients (38.0%) underwent IDS. The majority of patients (83.4%) had high-grade serous ovarian cancer, with a mean age of 61 ( ± 10) years at the time of adjuvant chemotherapy. Residual disease was present in 24.8% of patients (75.8% were R0). A total of 25 patients (18.3%) had a germline mutation in BRCA1 or BRCA2 (BRCAmut), 14 patients (10.2%) had a germline mutation in other genes (**Supplementary Table 1**), and 98 patients (71.5%) were WT. Seventy-seven patients (56.2%) received first-line platinum-based chemotherapy without bevacizumab and 60 patients (43.8%) with bevacizumab. Patient and tumor characteristics per type of surgery are listed in **Table 1**.

**Table 1:** Patient and tumor characteristics.

	All (n = 137)		Interval debulking surgery (n = 52)		Primary debulking surgery (n = 85)		*p*
	n	%	n	%	n	%	
Age at chemotherapy, years							0.013
Mean ± SD	61 ± 10		64 ± 10		59 ± 10		
Min–max	34–84		36–84		34–83		
Histology							0.108
High-grade serous	115	83.9	47	90.4	68	80.0	
Other histotypes	22	16.1	5	9.6	17	20.0	
Ascites							<0.001
No	73	53.7	10	19.6	64	75.3	
Yes	63	46.3	41	80.4	21	24.7	
Missing	1		1				
Post-surgical residual							0.969
R0	103	75.2	39	75.0	64	75.3	
R1+R2	34	24.8	13	25.0	21	24.7	
Type of chemotherapy							0.064
CT	77	56.2	24	46.2	53	62.4	
CTB	60	43.8	28	53.9	32	37.7	
Mutational status							0.781
BRCA mutant	25	18.3	10	19.2	15	17.7	
Other mutations	14	10.2	4	7.7	10	11.8	
Wild type	98	71.5	38	73.1	60	70.6	

CT, carboplatin and paclitaxel; CTB, carboplatin, paclitaxel, and bevacizumab.

With a median follow-up time of 67.9 months (95% CI 56.6–87.8) for the overall cohort, the median PFS was 22.5 months (95% CI 20.0–28.0) and the median OS was 81.4 months (95% CI 64.5–not reached (NR)). The median TTC was 45 days (1st–3rd quartile: 38; 58 days), and 21.9% of patients had a TTC ≥ 60 days. Among the covariates reported in **Table 1**, TTC was associated only with the type of surgery (33.6% of patients with TTC < 60 underwent IDS as compared to 53.3% of those with a longer TTC, *p* = 0.050) and residual disease (20.6% of patients with TTC < 60 had residual disease as compared to 40.0% of those with longer TTC, *p* = 0.029). Stratifying by type of surgery, we found an association between residual disease and TTC only in the subgroup of patients who received PDS. In particular, we found that among PDS patients with a TTC < 60 days, 12 (16.9%) patients had residual disease as compared to nine (64.3%) patients with a longer TTC, *p* < 0.001. Among IDS patients, 10 (27.8%) and three (18.8%) patients had residual disease in the subgroup with TTC < 60 days and TTC ≥ 60 days, respectively, *p* = 0.488.

In the entire study population, at univariate analysis, each day of delay in initiation of adjuvant chemotherapy resulted in an HR of 1.01 (95% CI, 1.00–1.02; *p* = 0.071) for PFS and an HR of 1.01 (95% CI, 0.99–1.02; *p* = 0.334) for OS. The hazard ratio of patients with a delay ≥ 60 days as compared to patients with a shorter delay was equal to 1.66 (95% CI 1.06–2.61, *p* = 0.026) for PFS and 1.44 (95% CI 0.78–2.64, *p* = 0.240) for OS. Other factors significantly associated with clinical outcomes were the type of surgery, PDS *vs.* IDS (HR = 0.48, 95% CI 0.33–0.71, *p* < 0.001, for PFS and HR = 0.28, 95% CI 0.16 –0.48, *p* < 0.001, for OS), presence of residual disease after surgery (HR = 2.37, 95% CI 1.54–3.64, *p* < 0.001, for PFS and HR = 1.79, 95% CI 1.01– 3.16, *p* = 0.045, for OS), and the presence of ascites (HR = 1.91, 95% CI 1.29–2.82, *p* = 0.001, for PFS and HR = 2.78, 95% CI 1.59– 4.86, *p* < 0.001, for OS). In an analysis stratified by residual disease, we found an association with a worse PFS but not OS only among patients with residual disease, even after adjusting for type of treatment (adjusted HR = 2.42 95% CI 1.02– 5.74, *p* = 0.044).

### 3.2 TTC in patients who underwent PDS

**Figure 2:**
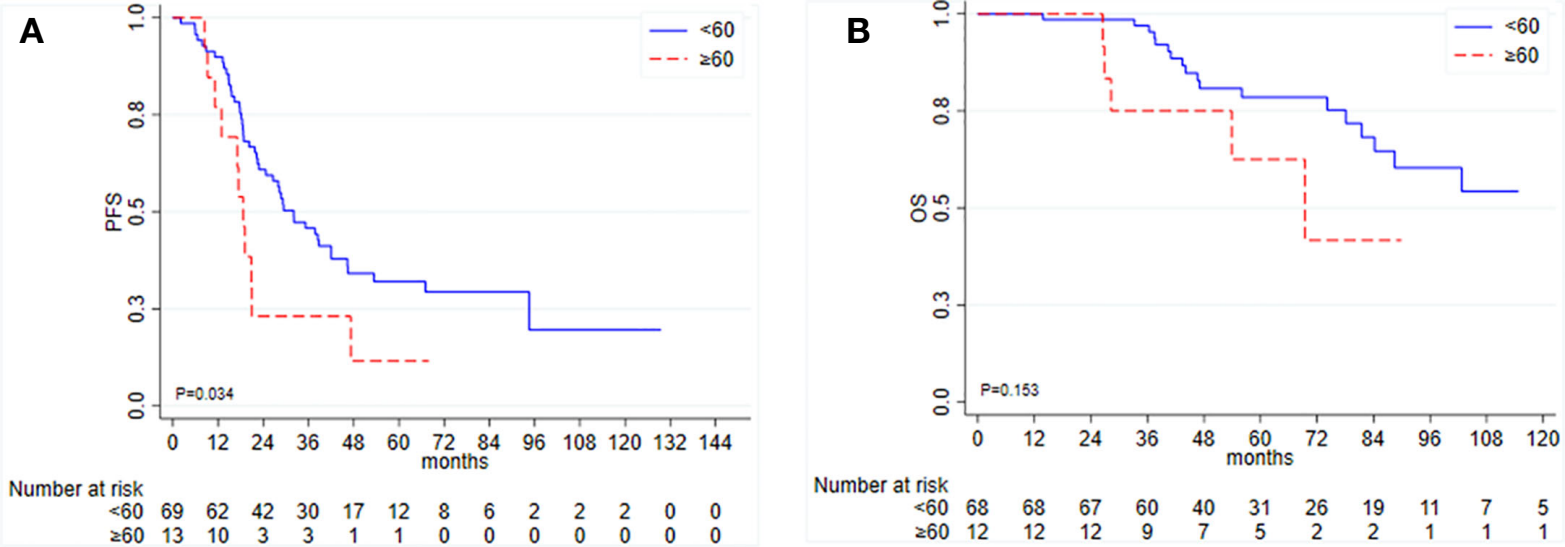
Kaplan–Meier curves for **(A)** progression-free survival (PFS) and **(B)** overall survival (OS) according to time to initiation of adjuvant chemotherapy (TTC) in patients undergoing primary debulking surgery.

Among the 83 patients (two patients were excluded for missing follow-up data) who underwent PDS, the median PFS was 28.8 months (95% CI 21.0–38.8), and the median OS was not reached (95% CI 84.2–NR). Patient and tumor characteristics per TTC are listed in **Table 2**. In univariate analysis, TTC ≥ 60 days was significantly associated with shorter PFS (HR = 2.02, 95% CI 1.04–3.93, *p* = 0.038) and worse OS (HR = 2.03, 95% CI 0.75–5.51, *p* = 0.161), although not statistically significant (**Figure 2**).

**Table 2:** Factors associated with the time to chemotherapy (TTC) in PDS patients.

	TTC			
	<60 (n = 71)	≥60 (n = 14)		
	n (%)	n (%)	*p*	
Age at surgery, years			0.967	
<61	41 (57.75)	8 (57.14)	
≥61	30 (42.25)	6 (42.86)	
Histology			0.726	
High-grade serous	56 (78.87)	12 (85.71)	
Other histotypes	15 (21.13)	2 (14.29)	
Ascites			1.000	
No	52 (73.24)	11 (78.57)	
Yes	19 (26.76)	3 (21.43)	
Post-surgical residual			<0.001	
R0	59 (83.10)	5 (35.71)	
R1+R2	12 (16.90)	9 (64.29)	
Mutational status			0.423	
BRCA mutant	11 (15.49)	4 (28.57)	
Other mutations	8 (11.27)	2 (14.29)	
Wild type	52 (73.24)	8 (57.14)	

The other factor significantly associated with PFS was the residual disease, with an HR of 2.97 (95% CI 1.67–5.26, *p* < 0.001) for R+ patients. Ascites, type of chemotherapy, and germline BRCA1/2 mutational status were not associated with PFS and thus were not included in the multivariate analysis. Among the 83 PDS patients, 16 (19.3%) received a PARP inhibitor as maintenance treatment: 10 (12%) in the second line and six (7.2%) in the third or fourth line.

In a multivariable model for PFS including TTC and residual disease as covariates, only the latter resulted in statistical significance: HR 1.52, 95% CI 0.75–3.06, *p* = 0.244, for TTC and HR 2.73, 95% CI 1.50–4.96, *p* = 0.001, for residual disease, respectively. In a stratified analysis by residual disease, there is a trend toward evidence of an association between TTC and PFS among patients with residual disease, n = 20 (HR = 2.53, 95% CI 0.86–7.47, *p* = 0.093). In the subgroup of patients with optimal debulking intervention (R0), this association was lost (HR = 1.04, 95% CI 0.32–3.39, *p* = 0.944). Both TTC ≥ 60 days and R+ were associated with worse OS (HR = 1.36, 95% CI 0.41–4.46; HR = 1.89, 95% CI 0.67–5.33), although not statistically significant.

### 3.3 TTC in patients who underwent IDS

**Figure 3:**
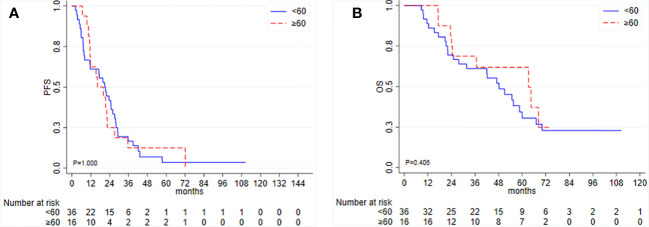
Kaplan–Meier curves for **(A)** progression-free survival (PFS) and **(B)** overall survival (OS) according to time to initiation of adjuvant chemotherapy (TTC) in patients undergoing interval debulking surgery.

Among the patients who underwent IDS, the median PFS was 20.0 months (95% CI 11.6–23.7), and the median OS was 54.8 months (95% CI 31.8–64.5). In this group, TTC was not associated with PFS or OS (**Figure 3**). Among 52 IDS patients, nine patients (17.3%) received a PARP inhibitor as a second-line maintenance treatment.

Again, ascites, residual disease, type of chemotherapy, and germline mutational status were not associated with clinical outcomes in IDS patients for either PFS or OS (data not shown).

## 4 Discussion

The exact time frame of chemotherapy initiation after surgery for advanced ovarian cancer remains unclear, and studies suggest that the worsening of clinical outcomes may be directly proportional to the passing of time (17). Similarly, a delay between the time of diagnosis (i.e., laparoscopy) and PDS undoubtedly increases patient anxiety and may also negatively impact survival. Surgery can cure some patients, but it also increases the risk of tumor spread more than diagnostic laparoscopy. For this reason, we chose to count the time to initiation of adjuvant chemotherapy from the definite surgery rather than from the diagnostic biopsy. However, surgical complexity and postoperative complications appear to be lower in patients undergoing IDS, which in a recent meta-analysis was shown to be similar to PDS in terms of OS and PFS (18).

In our analysis, we demonstrated that in advanced ovarian cancer patients who underwent PDS, the risk of a poorer prognosis is related to a TTC longer than the threshold value (>60 days), especially in those with residual disease, similar to the findings of Hofstetter and coworkers (8). On the contrary, Mahner and coworkers observed a small survival benefit only in patients with no residual disease after PDS (7). The FRANCOGYN research group found a non-significant trend in favor of a better PFS and a significantly higher OS in advanced ovarian cancer patients with a TTC below 8 weeks (19), whereas in early-stage (FIGO stage I–II) ovarian cancer, TTC was not associated with clinical outcomes (19, 20). Again, TTC affected the survival of stage IV ovarian cancer patients who underwent complete resection (21). Thus, TTC may be a prognostic factor in patients with advanced ovarian cancer where the presence of a microscopic residual disease after a complete cytoreduction surgery is more probable.

Macroscopic residual disease is the most important determinant of survival in ovarian cancer (22) also in our study. Perioperative complications associated with extended cytoreductive surgery can lead to prolonged postoperative convalescence, ultimately resulting in a delay in chemotherapy initiation. Surgical procedures (including in the upper abdomen, such as diaphragm resection, splenectomy, and cholecystectomy, all of which have their own individual risk of specific complications) necessary to obtain gross macroscopic radical resection increases the risk of peri- and postoperative complications and may for this reason prolong postoperative recovery, subsequently leading to a delay or even inhibition of adjuvant chemotherapy (23). This may be the reason why we found an association between longer TTC and the presence of residual disease. Therefore, it is difficult to independently evaluate these two closely related factors. It is likely that larger case studies will help answer this clinically relevant question. Recently, it was demonstrated that initiating adjuvant chemotherapy between 2 and 4 weeks after PDS with bowel resection (not before or after) may improve survival outcomes (24).

In order to avoid residual disease after surgery, efforts are needed to carefully select those patients with advanced ovarian cancer who would be better treated with PDS followed by chemotherapy than those who would benefit from neoadjuvant chemotherapy before IDS. In this context, laparoscopy before surgery has been shown to be effective for the evaluation of PDS candidate patients (25, 26). Furthermore, a diagnostic flowchart that integrates the use of a CT scan in combination with exploratory laparoscopy has proved to have superior diagnostic power when compared to CT alone in detecting the extent of the disease with particular regard to the diaphragm, the mesentery, and involvement of the small and large bowel (27, 28). Particularly for bowel resection, it allows you to plan the resection appropriately. It has been postulated that early initiation of chemotherapy in combination with antiangiogenic therapy is critical especially in patients with microscopic residual disease due to depletion of endogenous antiangiogenic factors (20). When we analyzed our data, we found an association with a higher risk of recurrence for patients with residual disease, regardless of the treatment they received (with or without antiangiogenic treatment).

More conflicting results were found in patients treated with IDS and the time to postoperative adjuvant chemotherapy, with data showing that decreasing TTC has prognostic relevance (24, 29). However, the time to IDS influenced the survival largely than the time to adjuvant postoperative chemotherapy. In the study conducted by Lee Y.J. and coworkers, indeed, TTC was not statistically significant, whereas the time from the last dose of neoadjuvant chemotherapy to surgery was statistically significant (24, 29). Thus, the time interval between the completion of neoadjuvant chemotherapy and the initiation of postoperative adjuvant chemotherapy may not be the best endpoint to consider. As for the TTC after PDS, the prolonged recovery time required by patients who undergo intensive surgery and postoperative complications are the main reasons for delays in treatment initiation after IDS. Although there are no chemotherapy-induced toxicities or postoperative complications, some clinicians prefer to schedule post-IDS adjuvant chemotherapy 3 weeks after IDS (24, 29).

However, in all these studies, mutational status was not taken into account, and other confounding factors may have influenced the results (17). Interestingly, mutational status was not associated with clinical outcomes in our analyses or with PDS or IDS patients. One of the possible explanations may be due to the relatively small casuistry. Another possible limitation that could have negatively influenced OS data in PDS patients might be related to the PARP inhibitor maintenance treatment in the second or later lines. Nonetheless, the number of patients who received a PARP inhibitor in the second line was really small (only 12%). Moreover, niraparib (a PARP inhibitor) maintenance therapy failed to show a significant difference in OS among patients with recurrent ovarian cancer, after adjusting for missing data burdens (25, 30). In this context, the treatment landscape of ovarian cancer changed radically in the last years with the introduction of maintenance therapy in the first line (26–28, 31–33). The use of neoadjuvant chemotherapy increased by approximately 10% per year from 2011 to 2016, without a change in the median survival trend, which increased by 2% per year (29, 34). However, the use of neoadjuvant chemotherapy increased also in non-high-grade serous histologies, although it was associated with a decreased OS compared with PDS in low-grade serous carcinoma (29, 34) and, for this reason, should be avoided. To assure adequate management of ovarian cancer patients, several factors need to be taken into account: contraindications to PDS related to tumor spread, patient-specific factors (e.g., co-existing illnesses, age, and WHO performance status (PS)), tumor histology, and mutational status. Thus, it will be very difficult in the future to evaluate the effect of TTC on advanced ovarian cancer patients. For this reason, we believe our work increases in importance because we analyzed TTC in the context of mutational status without first-line PARP inhibitor maintenance treatment.

## 5 Conclusions

It is highly probable that early initiation of adjuvant chemotherapy is not equally important for all ovarian cancer patients. Those treated with PDS may be worth treating as soon as possible, especially in patients with residual disease after surgery, as confirmed by our results on OS. However, before undergoing aggressive surgery, an appropriate preoperative assessment also becomes important that considers both the need to achieve R0 and an extensive surgery requiring a long recovery stay and consecutively a longer TTC. In our study, we have found suggestions for a possible time limit (60 days) that fits very well in routine clinical practice. However, time is a continuous variable, and we should always take into consideration that risk increases over time.

## Data availability statement

The raw data supporting the conclusions of this article will be made available by the authors, without undue reservation.

## Ethics statement

The studies involving humans were approved by AVR Ethics Committee (protocol 6326/2020). segreteriascientifica.ceav@irst.emr.it. The studies were conducted in accordance with local legislation and institutional requirements. The participants provided their written informed consent to participate in this study.

## Author contributions

Conceptualization: AF and UG. Methodology: AF and EP. Validation: UG, GT, and GG. Formal analysis: EP. Investigation: AF, AA, CC, CL, SB, GS, MR, and MS. Resources: CC, MR, VA, and MC. Data curation: AF, CC, MR, and VA. Writing— original draft preparation: AF. Writing— review and editing: AF, EP, CL, GS, GG, GT, and UG. Visualization: AF, GG, and GT. Supervision: UG. All authors have read and agreed to the published version of the manuscript.
