# Supplementary material for: Impact of the time interval between primary or interval surgery and adjuvant chemotherapy in ovarian cancer patients
Source: Front Oncol. 2023 Aug 16;13:1221096. doi: 10.3389/fonc.2023.1221096 (PMC10468566; doi:10.3389/fonc.2023.1221096)
Supplement: Supplementary file 1 [file Table_1.docx]

**Supplementary Table S1.** List of the 14 patients with mutations in genes other than *BRCA1/2* with the corresponding type of surgery.

| **Patient ID** | **Age at OC onset** | **Type of surgery** | ***BRCA1/2* status** | **Gene** | **Transcript** | **Exon** | **DNA (HGVS)** | **Protein (HGVS)** | **Variant type** | **IARC class*** | **dbSNP** | **ClinVar** |
| --- | --- | --- | --- | --- | --- | --- | --- | --- | --- | --- | --- | --- |
| A893 | 52 | primary debulking +/- HIPEC | wt | *PPM1D* | NM_003620 | 6 | c.1535dupA | p.Asn512LysfsTer16 | frameshift insertion | 4 | rs763475304 | - |
| A917 | 59 | interval surgery +/- HIPEC | wt | *PPM1D* | NM_003620 | 6 | c.1273delG | p.Asp425IlefsTer6 | frameshift deletion | 4 | - | - |
| B167 | 54 | interval surgery +/- HIPEC | wt | *MUTYH* | NM_012222 | 13 | c.1178G>A | p.Gly393Asp | missense variant | 5 | rs36053993 | pathogenic |
| B184 | 77 | primary debulking +/- HIPEC | wt | *EGFR* | NM_005228 | 7 | c.844G>T | p.Glu282Ter | nonsense variant | 4 | - | - |
|  |  |  |  | *CHEK2* | NM_007194 | 11 | c.1232G>A | p.Trp411Ter | nonsense variant | 5 | rs371418985 | pathogenic |
| B205 | 39 | primary debulking +/- HIPEC | wt | *ALK* | NM_004304 | 16 | c.2782dupT | p.Cys928LeufsTer20 | frameshift insertion | 4 | rs1218092221 | - |
| B330 | 70 | interval surgery +/- HIPEC | wt | *MITF* | NM_000248 | 9 | c.952G>A | p.Glu318Lys | missense variant | 5 | rs149617956 | pathogenic |
| B391 | 46 | interval surgery +/- HIPEC | wt | *MITF* | NM_000248 | 9 | c.952G>A | p.Glu318Lys | missense variant | 5 | rs149617956 | pathogenic |
| B406 | 69 | primary debulking +/- HIPEC | wt | *PALB2* | NM_024675 | 4 | c.1140_1143del | p.Ser380ArgfsTer43 | frameshift deletion | 5 | rs1257545151 | pathogenic |
| B426 | 78 | primary debulking +/- HIPEC | wt | *RAD51C* | NM_058216 | 1 | c.93delG | p.Phe32SerfsTer8 | frameshift deletion | 5 | rs730881942 | pathogenic |
| B476 | 45 | primary debulking +/- HIPEC | wt | *MUTYH* | NM_012222 | 7 | c.527A>G | p.Tyr176Cys | missense variant | 5 | rs34612342 | pathogenic |
| B513 | 54 | primary debulking +/- HIPEC | wt | *RAD51C* | NM_058216 | 7 | c.905-2_905-1del | p.? | splicing variant | 5 | rs587781995 | pathogenic |
| B618 | 68 | primary debulking +/- HIPEC | wt | *MUTYH* | NM_012222 | 13 | c.1178G>A | p.Gly393Asp | missense variant | 5 | rs36053993 | pathogenic |
| B693 | 43 | primary debulking +/- HIPEC | wt | *MLH1* | NM_000249 | 12 | c.1039-1G>C | p.? | splicing variant | 4 | rs267607819 | likely-pathogenic |
| B697 | 54 | primary debulking +/- HIPEC | wt | *MITF* | NM_000248 | 9 | c.952G>A | p.Glu318Lys | missense variant | 5 | rs149617956 | pathogenic |

* IARC class: 5 - pathogenic; 4 - likely-pathogenic
